# Enhancing graft stability in coronally advanced tunnel with connective tissue graft for Cairo RT1 and RT2 gingival recession: a case series

**DOI:** 10.1093/jscr/rjag390

**Published:** 2026-05-24

**Authors:** Dler Ali Khursheed

**Affiliations:** Department of Periodontics, College of Dentistry, University of Sulaimani, Madame Mitterrand Street, Sulaymaniyah 46001, Kurdistan Region, Iraq

**Keywords:** gingival recession, coronally advanced tunnel, connective tissue graft, root coverage, periodontal plastic surgery

## Abstract

Gingival recession (GR) in the mandibular anterior region presents a clinical challenge because factors such as shallow vestibular depth, frenal pull, and limited soft tissue thickness may compromise graft stability during root coverage procedures. This case series describes the management of Cairo RT1 and RT2 GR defects using a modified coronally advanced tunnel technique combined with subepithelial connective tissue grafting and tooth-anchored encircling sutures for graft stabilization. Five systemically healthy patients presenting with eleven recession defects were treated. At the 3-month follow-up, a high level of root coverage was achieved, with complete root coverage observed in most sites, including all RT1 defects and the majority of RT2 defects. Increased gingival thickness was also observed in several treated sites. The modified technique, emphasizing graft stabilization and passive flap mobility, may enhance clinical outcomes in mandibular anterior GRs.

## Introduction

Gingival recession (GR), defined as the apical displacement of the gingival margin relative to the cementoenamel junction (CEJ), is a common mucogingival condition associated with dentin hypersensitivity, aesthetic concerns, and increased risk of root caries [1, 2]. The Cairo classification provides prognostic stratification of recession types (RT), with RT1 defects showing more predictable root coverage outcomes than RT2 defects [2, 3].

The mandibular anterior region represents a particularly challenging area for root coverage therapy because of anatomical factors such as thin periodontal phenotype, shallow vestibular depth, and high frenal attachment. The latter two factors may increase tension on the surgical wound and compromise graft stability during early healing [4–7].

The modified coronally advanced tunnel technique (MCAT) with connective tissue graft (CTG) has been widely used for root coverage procedures because it preserves interdental papillae and maintains vascular supply while avoiding vertical releasing incisions [8–10]. However, graft micromotion may still occur in sites with increased muscular traction.

While the coronally advanced tunnel technique combined with CTG has been widely reported, limited attention has been given to strategies specifically aimed at controlling graft micromotion during early healing. In particular, active graft stabilization techniques and their potential influence on clinical outcomes remain insufficiently explored.

Therefore, the aim of this case series was to describe a MCAT technique with particular emphasis on graft stabilization and to evaluate its clinical outcomes in Cairo RT1 and RT2 GR defects.

## Case series

### Patient characteristics and clinical presentation

Five patients presenting with GR defects in the mandibular anterior region were included in this case series. The cohort consisted of four females and one male, aged between 23 and 38 years. All patients were systemically healthy, nonsmokers, and maintained good oral hygiene following initial periodontal therapy.

Clinical examination identified 11 recession defects involving mandibular incisors and canines. Recession depth was measured from the cementoenamel junction to the gingival margin using a UNC periodontal probe, and gingival phenotype was assessed using the probe transparency method.

According to the Cairo classification, five defects were classified as RT1 and six as RT2. Most sites presented with a thin gingival phenotype.

All patients underwent initial periodontal therapy, followed by surgical treatment after a 3-month re-evaluation period.

Detailed baseline characteristics are presented in Table 1.

**Table 1:** Baseline characteristics and clinical outcomes of mandibular anterior GR defects treated using a MCAT technique with connective tissue graft stabilization.

Case	Age	Sex	Tooth	RT	RD baseline (mm)	RW (mm)	RD 3 months (mm)	% Root coverage	Keratinized width	Phenotype baseline	Phenotype 3 months	Outcome
1	23	F	31	RT1	6	3	0	100	Narrow	Thin	Thin	CRC
2	25	M	31	RT1	6	1	0	100	Wide	Thick	Thick	CRC
3	33	F	33	RT1	1	4	0	100	Wide	Thin	Thick	CRC
			32	RT1	2	3	0	100	Wide	Thin	Thick	CRC
			31	RT2	4	3	1	75	Narrow	Thin	Thin	PRC
			41	RT2	4	3	2	50	Narrow	Thin	Thin	PRC
			42	RT1	1	3	0	100	Wide	Thin	Thick	CRC
4	33	F	31	RT2	2	4	0	100	Wide	Thin	Thick	CRC
			41	RT2	3	3	0	100	Wide	Thin	Thick	CRC
5	38	F	32	RT2	2	3	0	100	Narrow	Thin	Thick	CRC
			31	RT2	1	3	0	100	Narrow	Thin	Thick	CRC

Measurements include recession depth (RD) at baseline and 3-month follow-up, recession width (RW), gingival phenotype, keratinized tissue (KT) width, and percentage of root coverage. Outcomes are categorized as complete root coverage (CRC) or partial root coverage (PRC).

### Surgical technique

All procedures were performed under local anaesthesia using a microsurgical approach and magnification. Flap preparation was carried out exclusively with tunnelling instruments from the initial intrasulcular access to completion of the tunnel dissection [11].

### Recipient site preparation

The exposed root surfaces within the surgical field were mechanically debrided using manual scalers and Gracey curettes to achieve a clean and biologically compatible recipient surface prior to surgical intervention.

Intrasulcular incisions were performed at the involved teeth without vertical releasing incisions. A full thickness mucoperiosteal tunnel was initially developed along the root surfaces and underlying alveolar bone using MEDESY® tunnelling instruments (698/1.HL8, 698/2.HL8, 698/3.HL8). This dissection allowed mobilization of the soft tissue complex and elevation of the periosteum from the bone while maintaining papillary integrity [8].

**Figure 1:**
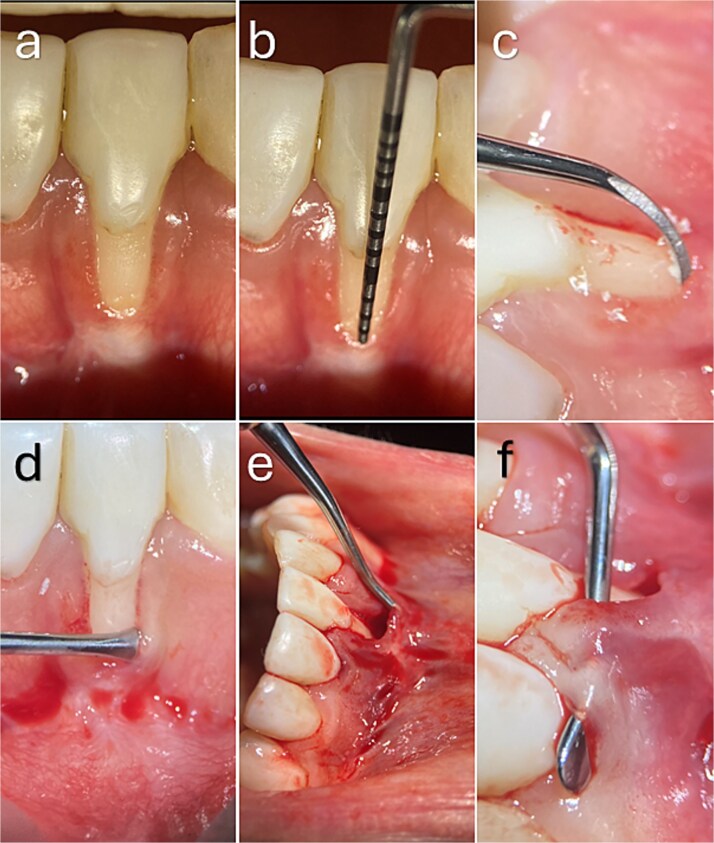
Recipient site preparation and tunnel development. (a, b) Baseline clinical presentation showing a buccal gingival recession defect in the mandibular anterior region associated with a thin periodontal phenotype and reduced keratinized tissue, without interproximal attachment loss. (c) Root surface instrumentation. (d–f) Tunnel preparation using tunnelling instruments, extending beyond the mucogingival junction and laterally beneath adjacent papillae while preserving papillary integrity and vascular supply.

The tunnel was extended laterally beneath the adjacent papillae up to their distal aspects to preserve interdental vascular supply (Fig. 1). Interdentally, the papillae were carefully released toward the lingual aspect without incising the papillary tips in the RT2, thereby increasing flap mobility and facilitating coronal advancement while preserving papillary continuity and vascularization (Fig. 5e). Apically, the full-thickness dissection was advanced to the level of the mucogingival junction.

To facilitate passive coronal advancement of the flap, the tunnelling instrument was then directed superficially beyond the mucogingival junction, and the dissection was continued in a partial thickness supraperiosteal plane, separating the alveolar mucosa from the underlying tissues and increasing flap mobility.

In sites presenting with shallow vestibular depth or high frenal attachment, additional blunt apical release was performed to disengage muscular and frenal insertions and reduce tractional forces on the surgical site. This manoeuvre allowed tension-free coronal positioning of the flap and minimized destabilizing forces that could compromise graft stabilization during early healing.

### Connective tissue graft harvesting

CTGs were harvested from the palatal donor sites. The grafts were extraorally de-epithelialized and trimmed to a uniform thickness of ~1.0–1.5 mm to ensure optimal adaptation to the recipient bed and extension beneath the adjacent papillae (Supplementary Video 1).

### Graft insertion and stabilization

**Figure 2:**
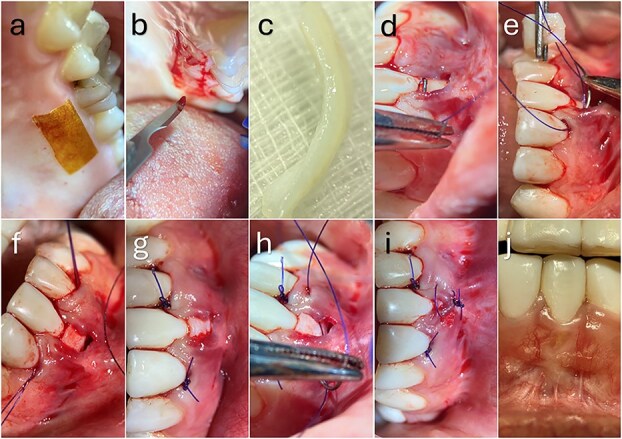
Connective tissue graft harvesting, insertion, stabilization, and clinical outcome. (a–c) Harvesting of the connective tissue graft from the palatal donor site and extraoral de-epithelialization to obtain a uniform graft thickness. (d–f) Suture-guided insertion and positioning of the graft within the tunnel to ensure intimate adaptation to the root surface and recipient bed. (g) Graft stabilization using a tooth-anchored encircling suture to minimize micromotion during early healing. (h, i) Passive coronal advancement of the flap and tension-free closure. (j) Clinical outcome at 3 months demonstrating complete root coverage and improved gingival tissue contour.

The CTG was inserted into the prepared tunnel using a suture-guided delivery technique. A tooth-anchored encircling sling suture was then placed around the adjacent teeth to secure the graft against the root surface and stabilize the graft–flap complex (Fig. 2). This stabilization approach was intended to maintain intimate graft adaptation to the recipient bed and reduce graft micromotion during the early healing phase.

### Flap advancement and closure

Following stabilization of the lateral aspects of the graft, the marginal tissues were passively advanced in a coronal direction to achieve complete coverage of the CTG. Adequate tunnel extension allowed tension-free flap positioning without exerting compressive forces on the underlying graft. Interrupted sutures were strategically placed at the mid-buccal gingival margins in RT2 sites to further secure the graft–flap complex.

The sutures were secured to the corresponding crowns using flowable composite. This approach enhanced graft immobilization, optimized flap adaptation, and preserved vascular supply from both the periosteal bed and the overlying flap, thereby supporting early wound stability and revascularization.

### Postoperative care

Patients were prescribed systemic antibiotics and analgesics, including amoxicillin–clavulanic acid (1 g twice daily) and mefenamic acid (500 mg three times daily). Patients were instructed to avoid mechanical plaque control at the surgical site for 2 weeks and to rinse twice daily with 0.12% chlorhexidine gluconate.

Sutures were removed after 14 days, and healing was monitored through scheduled follow-up visits.

## Case descriptions

### Case 1

A 23-year-old female presented with a Cairo RT1 GR defect at tooth 31 with a baseline recession depth of 6 mm and a thin periodontal phenotype. Following treatment with the MCAT technique, complete root coverage was achieved at 3 months, with improved gingival thickness and soft tissue contour (Fig. 2j).

### Case 2

**Figure 3:**
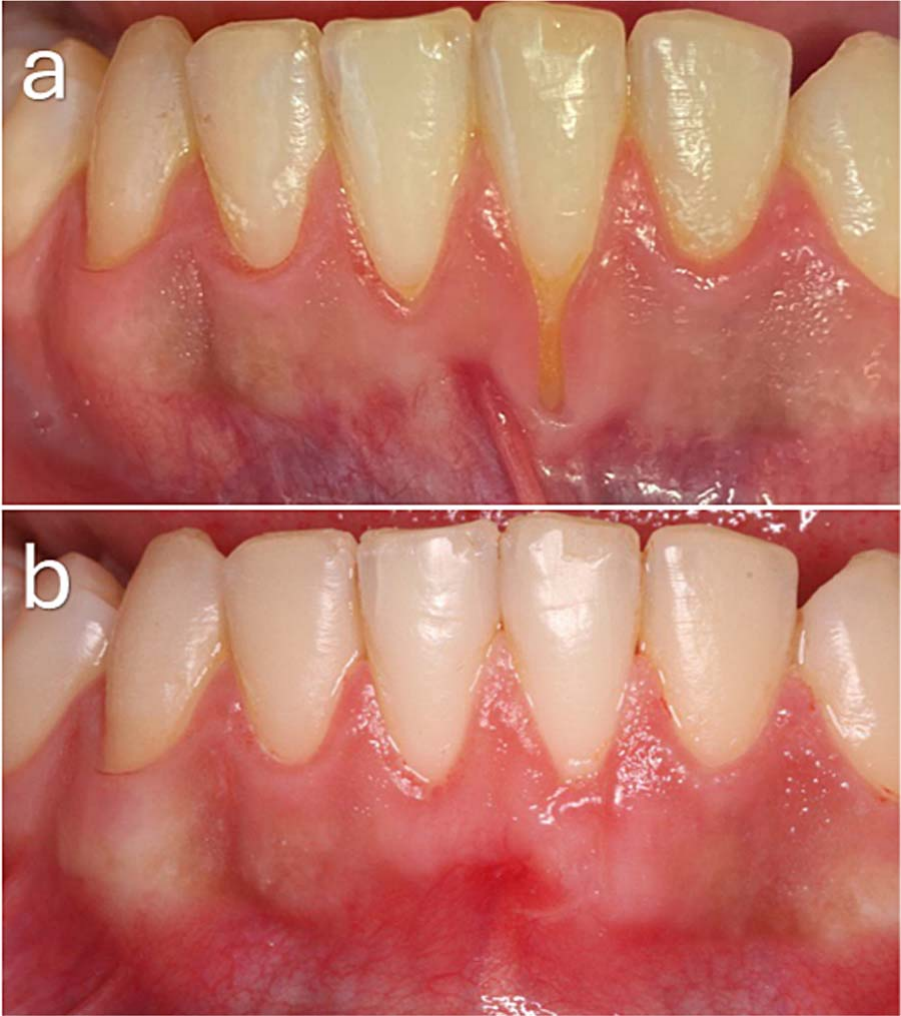
Baseline and 3-month clinical views demonstrating complete root coverage following treatment of a Cairo RT1 gingival recession.

A 25-year-old male presented with a Cairo RT1 GR defect at tooth 31 with a baseline recession depth of 6 mm and a thick phenotype. Complete root coverage was observed at 3 months, with stable marginal tissue position (Fig. 3).

### Case 3

**Figure 4:**
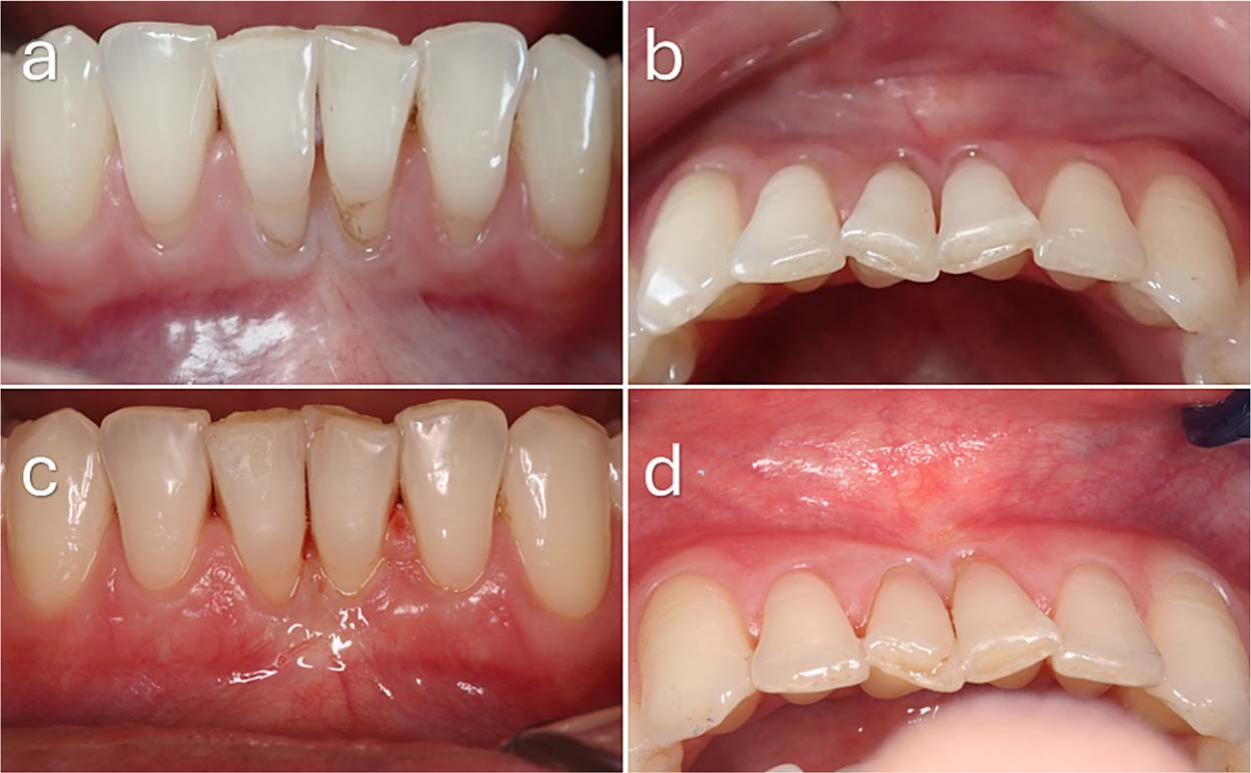
Baseline and 3-month clinical outcomes following mucogingival surgery. (a, b) Preoperative clinical views demonstrating gingival recession in the mandibular anterior region, including Cairo RT1 defects at the lateral incisors and left canine, and RT2 defects at the central incisors. A thin periodontal phenotype with reduced gingival thickness and limited keratinized tissue width is evident. (c, d) Clinical appearance at 3 months postoperatively. Complete root coverage was achieved at RT1 sites, whereas partial root coverage was observed at RT2 sites. An increase in gingival thickness and improved soft tissue contour are also evident.

A 33-year-old female presented with multiple GR defects involving teeth 33, 32, 31, 41, and 42. RT1 defects demonstrated complete root coverage, whereas RT2 defects at teeth 31 and 41 showed only partial root coverage. Overall, an increase in gingival thickness was observed across treated sites (Fig. 4).

### Case 4

**Figure 5:**
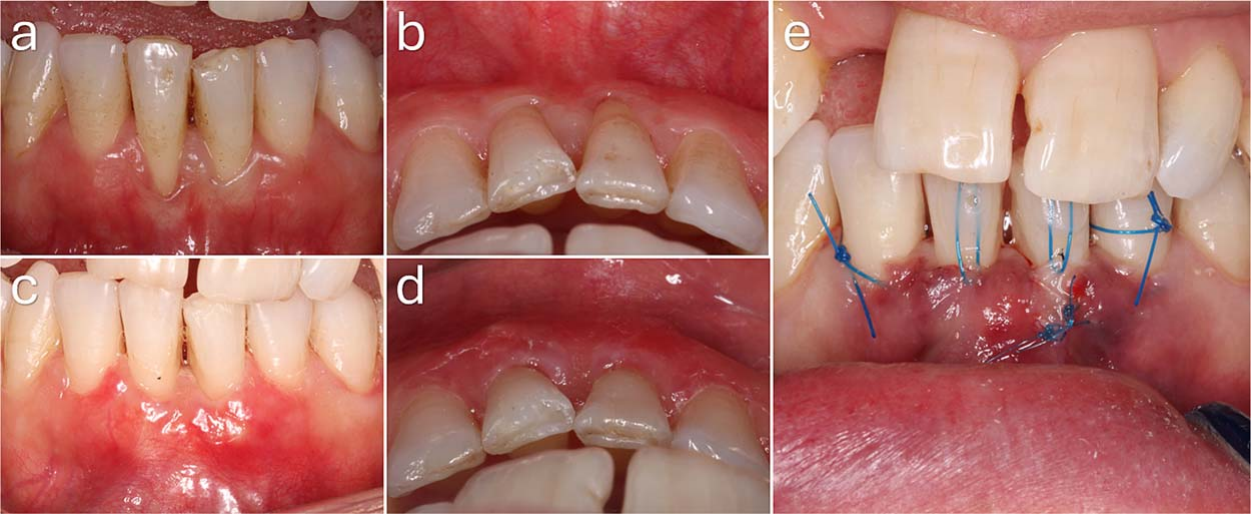
Baseline and 3-month clinical outcomes of gingival recession defects in the mandibular anterior region. (a, b) Preoperative frontal and occlusal views demonstrating Cairo RT2 gingival recession at the mandibular central incisors, associated with a thin periodontal phenotype and reduced keratinized tissue width. (c, d) Clinical appearance at 3 months postoperatively showing complete root coverage with increased gingival thickness and improved soft tissue contour. (e) Intraoperative view demonstrating interdental papillary extension with lingual release to enhance flap mobility and facilitate coronal advancement of the graft–flap complex.

A 33-year-old female presented with Cairo RT2 GR defects at teeth 31 and 41, associated with a thin phenotype. Complete root coverage was achieved at both sites at 3 months, with improved soft tissue thickness and contour (Fig. 5).

### Case 5

**Figure 6:**
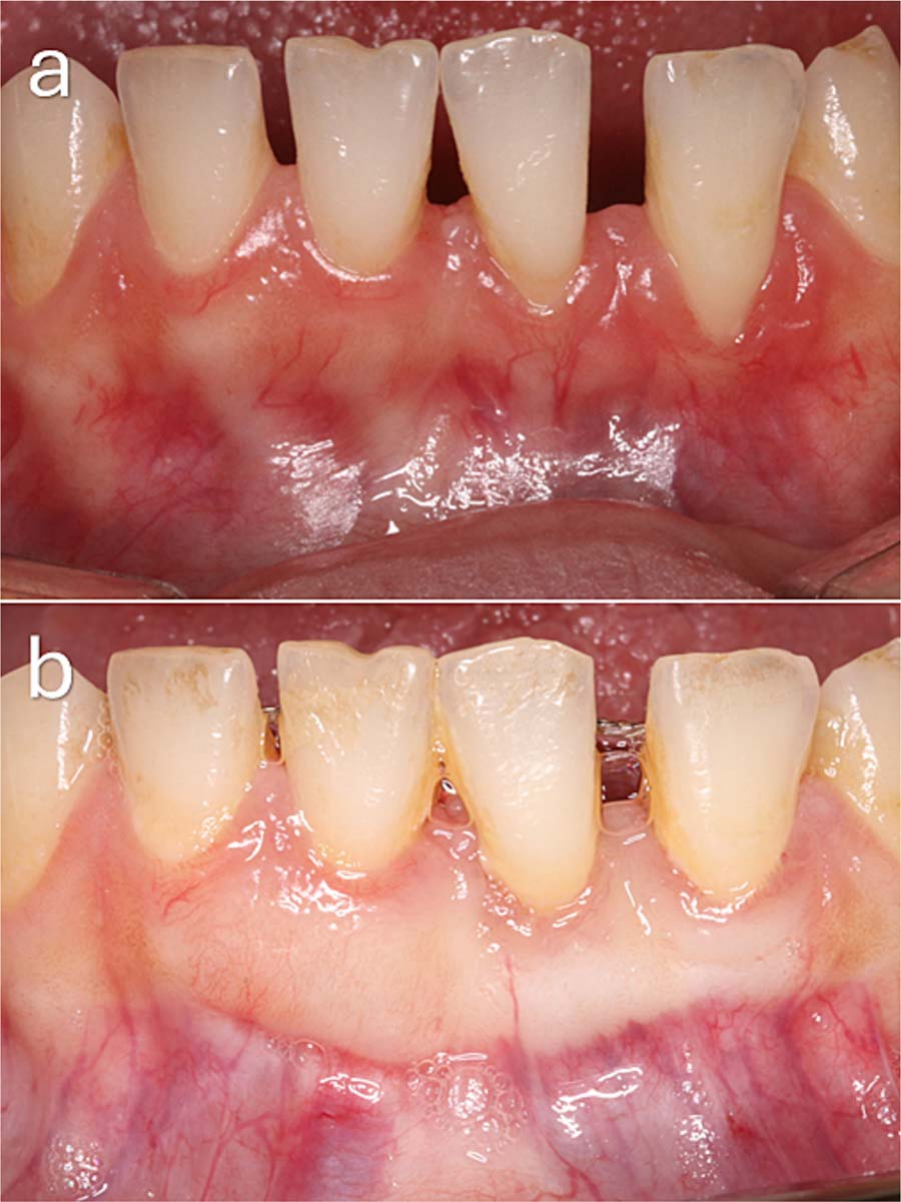
Baseline and 3-month clinical outcomes following a modified coronally advanced tunnel procedure in the mandibular anterior region. (a) Preoperative clinical view demonstrating gingival recession at teeth 31 and 32 associated with a thin periodontal phenotype and deep vestibular depth. Incisal attrition and spacing between teeth 31 and 32 are evident. (b) Clinical appearance at 3 months postoperatively showing complete root coverage with increased gingival thickness and improved soft tissue contour.

A 38-year-old female presented with Cairo RT2 GR defects at teeth 31 and 32, with baseline recession depths ranging from 1 to 2 mm. Complete root coverage was achieved at both sites, with enhanced gingival thickness at 3 months (Fig. 6).

## Discussion

This case series demonstrates favourable short-term outcomes following treatment of mandibular anterior GR using a MCAT technique with enhanced graft stabilization. A high level of root coverage was achieved, with complete root coverage in the majority of sites and a mean root coverage of 93.2%, as detailed in Table 1. These findings are consistent with recent studies demonstrating that the combination of MCAT and CTG provides predictable root coverage and soft tissue augmentation [12].

The findings highlight the influence of defect characteristics on treatment outcomes. While all RT1 defects achieved complete root coverage, RT2 defects showed reduced predictability [3]. Nevertheless, most RT2 sites achieved complete root coverage, and the remaining sites demonstrated clinically meaningful partial root coverage (50%–75%). Similar observations have been reported in recent clinical trials, where MCAT combined with CTG achieved stable outcomes in both RT1 and RT2 defects over time [13]. The reduced predictability observed in RT2 defects may be partly explained by increased functional tension within the recipient site, particularly in areas with limited vestibular depth and enhanced muscular pull. These factors may promote graft micromotion during early healing, thereby compromising graft stability and revascularization.

The present modification differs from conventional MCAT approa-ches by incorporating active graft stabilization strategies, rather than relying solely on flap design. In particular, the use of tooth-anchored encircling sutures, supplemented by additional coronal fixation, allows controlled immobilization of the graft–flap complex during early healing. This shift from passive to active stabilization may represent an important factor in improving clinical outcomes, particularly in RT2 defects.

A critical factor influencing the success of root coverage procedures is the stability of the graft during the early healing phase [14]. Excessive graft micromotion may disrupt fibrin clot adhesion and impair revascularization, thereby compromising graft integration. Ensuring intimate adaptation of the CTG to the recipient bed facilitates clot stabilization and provides a favourable biological environment for angiogenesis and tissue integration [12].

In addition, the combined full-thickness and supraperiosteal tunnel preparation, together with controlled papillary extension—including lingual release—enabled passive, tension-free coronal advancement of the flap. Tunnel-based approaches have been shown to preserve vascular supply and provide outcomes comparable to or better than conventional coronally advanced flap techniques [15, 16]. Preservation of dual blood supply from both the periosteal bed and the overlying flap may further enhance graft survival and integration.

An increase in gingival thickness was observed in several sites, with conversion from thin to thick phenotype following connective tissue grafting. This finding is in agreement with previous studies demonstrating the role of CTG in soft tissue augmentation and phenotype modification, which may contribute to long-term stability of root coverage outcomes [12, 17].

The limitations of this report include the small sample size and the relatively short follow-up period.

## Conclusion

The MCAT technique with tooth-anchored graft stabilization demonstrated favourable short-term outcomes for mandibular anterior GR defects. The approach provided predictable results in RT1 defects and encouraging outcomes in RT2 defects, together with improvement in soft tissue thickness. This technique may enhance graft stability in challenging mucogingival conditions.

## Supplementary Material

rjag390_Supplemental_Files

## Clinical and surgical implications

Enhanced graft stabilization using tooth-anchored encircling sutures and controlled tunnel extension may improve the predictability of root coverage, particularly in RT2 defects. Minimizing graft micromotion and achieving passive, tension-free flap advancement appear critical for stable healing and successful clinical outcomes.
